# SF-1 mediates reproductive toxicity induced by Cerium oxide nanoparticles in male mice

**DOI:** 10.1186/s12951-019-0474-2

**Published:** 2019-03-21

**Authors:** Fenju Qin, Tao Shen, Jinlin Li, Junchao Qian, Jie Zhang, Guangming Zhou, Jian Tong

**Affiliations:** 1 School of Chemistry, Biology and Material Engineering, Suzhou University of Science and Technology, Suzhou, 215009 China; 2 School of Public Health, Medical College of Soochow University, Suzhou, 215123 China; 3 School of Radiation Medicine and Protection, Medical College of Soochow University, Suzhou, 215123 China

**Keywords:** CeO_2_ NPs, Reproductive toxicity, Testosterone, Steroidogenesis genes, SF-1

## Abstract

**Background:**

Cerium oxide nanoparticles (CeO_2_ NPs) have potential application for use in biomedical and in various consumer products. However, it is largely unclear whether CeO_2_ NPs have effects on male reproductive function.

**Methods:**

In this study, male mice were examined for toxicity, if any, following chronic oral administration of CeO_2_ NPs for 32 days. In each animal, epididymides were examined for sperm motility and DNA integrity. Bloods were tested for testosterone levels. Testicular tissues were collected to determine the element Ce content, the daily sperm production (DSP), marker enzymes such as ACP, G6PD, γ-GT and SDH, mRNA expression levels of steroidogenesis genes *Star, P450scc, P450c17, 3β*-*Hsd,* and *17β*-*Hsd*, as well as steroidogenic factor-1 (SF-1) gene/protein levels.

**Results:**

The results showed that CeO_2_ NPs (20 mg/kg and 40 mg/kg) increased the element Ce content in testis, the testis histopathological patterns and sperm DNA damage whereas decreased the testis weight, DSP and sperm motility. There were also remarkable reduction in testosterone levels and marker enzymes activities, down-regulated mRNA expression levels of several steroidogenesis genes such as *Star, P450scc, P450c17, 3β*-*Hsd,* and *17β*-*Hsd*, as well as altered gene and protein expressions of SF-1.

**Conclusion:**

These results reveal the male reproductive toxicity of chronic exposure of CeO_2_ NPs in mice, hinting that the utilization of CeO_2_ NPs need to be carefully evaluated about their potential reproductive toxicity on the human health.

**Electronic supplementary material:**

The online version of this article (10.1186/s12951-019-0474-2) contains supplementary material, which is available to authorized users.

## Background

There are more than 1814 different nanomaterials that are currently used in consumer products because of their unique properties such as magnetism, thermotics, and optics [1, 2]. The potential health risk of engineered nanomaterials has been a great concern of the general public, but only 3% of the investigation on nanoparticles (NPs) is devoted to their biological effects [3, 4]. Lack of adequate knowledge of the adverse effects of nanomaterials, together with intentional therapeutic use and unintentional environmental contamination, has exposed the public from unperceived health risks. The extremely small size of nanoparticles enable them cross biological membranes and enter into the cell [5, 6]. Getting into the circulatory system by inhalation, ingestion, or penetration through the skin, NPs can be distributed to other organs and tissues in the body to induce adverse effects such as oxidative stress, activation of inflammatory cytokines, gene mutations and even cell death [7, 8].

The existing knowledge on potential adverse effects of NPs on reproductive system is not complete. There were some reports that the metal NPs could be found in animal testicles and ovaries after in vivo exposure [9, 10]. Among the various nanomaterial products, cerium oxide (CeO_2_), a lanthanide element oxide, is widely used in industry processing such as benzene degradation, manufacture of solar/fuel cells, UV absorbents, oxygen sensors, oxygen pumps, polishers for chemical mechanical planarization, metallurgical, ceramic and smart glass, etc. [11–13]. As a result, the numbers of workers exposed to CeO_2_ nanoparticles increase rapidly. There are studies which have investigated the bio-distribution, bio-accumulation of CeO_2_NPs in vivo and the toxicity in lung, liver and blood, even DNA damage [14–16]. However, the impact of CeO_2_ NPs on reproductive function, particularly on male reproductive system, remains poorly understood. The experimental results from available reports so far in scientific literature were largely controversial. Some studies showed a remarkable increase in DNA damage in mouse sperm induced by CeO_2_ NPs even at concentrations as low as 0.01 mg/L [17]. In vitro exposure of human spermatozoa to CeO_2_ was also reported to induce cellular damage due to the accumulation of nanoparticles on the plasma membranes [18]. In contrast, short-term in vitro exposure of CeO_2_ NPs did not induce changes in ram sperm function and morphology [19]. CeO_2_ NP was also reported to be found in the testicles and epididymis in rats after inhalation, but the reproductive outcomes associated with CeO_2_ NP accumulation in testis were not assessed [20]. Overall, the available information on the male reproductive toxicity of CeO_2_ NP is very limited and incomplete [21].

In the present study, the possible impact of chronic exposure to CeO_2_NP on the element Ce content in testis, sperm parameters, integrity of the DNA, histopathology, testis marker enzymes, testosterone levels, and expression of genes involved in steroidogenesis were evaluated, in order to explore potential health effects of chronic administration of CeO_2_NP on the male reproductive function.

## Materials and methods

### Animal handling

Adult male 6 week-old C57BL/6J mice weighing 22 ± 2 g were obtained from the laboratory animal center of Soochow University. They were maintained in strict accordance with the Institutional Animal Care and Use Committee guidelines of the University. The animals were housed in a facility maintaining 25 + 2 °C temperature, 50 + 5% relative humidity and 12 h light/dark cycles. They were fed with commercial diet and provided water ad libitum. After 7 days of quarantine, the animals were randomly divided into one control group and three experimental groups, each with 12 mice. The 3 experimental groups were given oral administration of CeO_2_ NPs (Sigma-Aldrich, Shanghai, China) at doses of 10, 20 or 40 mg/kg body weight, respectively, for 32 consecutive days at 9:00 AM every day. Nanoparticles were suspended in 0.50% solvent (carboxymethylcellulose sodium salt, Sigma-Aldrich, China) by ultrasonication and vortexed before every treatment. The control mice were given the same volume of 0.50% solvent at the same time each day. In the thirty-third day, all mice were sacrificed by cervical dislocation. From each animal, blood was collected by periorbital puncture into heparinized tubes and plasma was separated for determination of testosterone using ELISA kit. The sperm was collected from epididymis to examine the motility and DNA integrity. The right testis randomized from 6 mice in each group was excised, fixed and tissue sections were used for histological evaluation, the left testis was kept frozen at − 80 °C for ICP-MS analysis. For the testis from the other 6 mice in each group, the right was immediately used to test testicular enzymes activities and sperm head count, the left was kept frozen until used for real-time PCR or Western blot test.

### Nanoparticles

The cerium oxide nanoparticles (Product Number: 544841, APS: < 25 nm and purity > 99% trace metal basis) were obtained from Sigma-Aldrich (Shanghai, China). Their characteristics were tested by X-ray powder diffractometry (XRD8 Advance X-ray diffracto

**Fig. 1:**
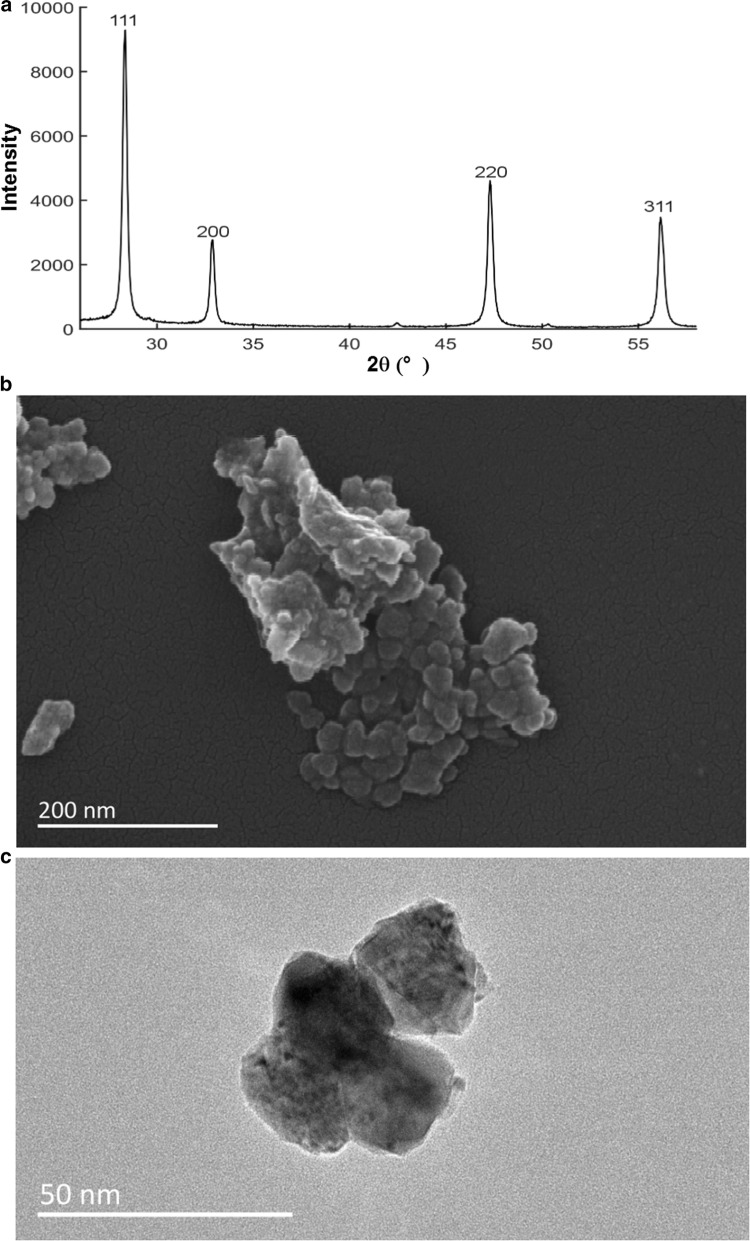
Characterization of test material CeO_2_ NPs. **a** The results of XRD test; **b** scanning electron micrographs of CeO_2_; **c** transmission electron micrographs of CeO_2_

-meter, Bruker AXS Endeavor, Billerica, USA), SEM (Quanta FEG 250, Hillsboro, USA) and transmission electron microscope (TEM, JEOL 2100, Tokyo, Japan) and presented in Fig. 1a–c. The X-ray diffraction of the precipitated material showed cubic crystals. The intense peaks from the XRD test corresponded to the diffraction peak of CeO_2_. The average size of CeO_2_NP evaluated by TEM was 27.62 ± 3.01 nm.

### ICP-MS analysis

The frozen testis was thawed, weighed and soaked in 10 ml concentrated nitric acid overnight. Then the sample was digested in graphite digestion furnace at 300 °C until transparent. Digestion liquid was heated and vaporized to 1.5 ml. After cooling, the samples were diluted with Milli-Q water to 10 ml. The Cerium concentrations were measured using ELEMENT 2 ICP-MS (Thermo Fisher, United States) [22].

### Histopathology

Histological sections of the testicular tissue (5 μm) were prepared and stained with hematoxylin and eosin [23]. For each animal, 3 sections at random were examined for microscopic architectures and photographed using a fluorescence microscope (Zeiss, Germany). The extent of damage was evaluated by the percentage of degenerated tubules [24].

### Measurement of testosterone and marker enzymes

For each animal, the concentration of testosterone in plasma was measured by ELISA kit (Shanghai Yili, China) according to the protocols from the manufacturer. The sensitivity of the assay ranged from 8 to 240 nmol/l. The assay in each sample was repeated three times.

The testis from each mouse was homogenized in 0.9% normal saline containing 0.05% Triton X-100 (Sigma-Aldrich, China). After centrifugation, the supernatant was used to measure the levels of four testis marker enzymes, viz., acid phosphatase (ACP), glucose-6-phosphate dehydrogenase (G6PD), γ-glutamyl-transpeptidase (γ-GT) and succinate dehydrogenase (SDH) using commercially available kits (Suzhou Comin Biotechnology Co., Ltd, China).

### Daily sperm production (DSP)

The number of sperm produced per gram testicular tissue every day was detected and calculated as described by Joyce et al. [25]. Briefly, testes were weighed, decapsulated, and homogenized in ice-cold physiological saline solution containing 0.01% Triton X-100. The homogenate was defoamed by 1 min settling and was gently mixed and stored on ice. An aliquot of the homogenate was used to evaluate the number of spermatozoa with a hemocytometer for 14–16 spermatids (stages II–VIII) surviving after this homogenization. The total spermatids value per testis was divided by testis weight (g) and the time (days) during spermatogenesis to obtain the daily sperm production (DSP). It is worth noting that the time (days) during spermatogenesis is 4.84 days for mice [26]. Each sample was examined three times and averaged.

### Sperm motility and DNA integrity

For each mouse, the left epididymis was used to obtain a free sperm suspension by mincing in warm Hank’s solution (Ca^2+^ and Mg^2+^ free). The motility of sperm was classified according to instructions in WHO (1992). Each sample was examined three times.

Sperm DNA integrity was determined by SCSA using acridine orange (AO) staining kit (Hezhong biotechnology co., Ltd, China) to distinguish the sperm metachrome shift according to change in fluorescence from green to orange-red (undenatured DNA to denatured DNA) as described by Duale et al. [27]. A small aliquot of sperm suspension from the right epididymis was washed and centrifuged. The cell pellet was diluted, resuspended with dilution solution and 15 μl sperm suspension was added on a microscope slide, 2 ml fixative was added after drying, and then, fixed for 5 min and stained with AO staining solution for 15 min at 37 °C protected from light. Then staining solution was washed and the slides were covered with glycerol medium. The smear with AO staining was examined and photographed using a fluorescence microscope (Zeiss, Germany). The green fluorescence color reflects the sperm with normal double-stranded DNA while the red fluorescence color denoted denatured sperm DNA. In each sample, 200 sperm were examined to record the percentage with red fluorescence sperms.

### Real-time PCR

Total RNA extraction of each testis was conducted using High Pure RNA kit from Roche, Switzerland. Then NanoDrop™ 2000C was used to detect the RNA concentration at 260 nm. The reverse transcription and real-time PCR was carried out as described earlier [28]. The sequences of the primers (*Sf*-*1, StAR, P450scc, P450c17, 3β*-*HSD, 17β*-*HSD*) used are shown in the following. The 2^−ΔΔCt^ method was used to calculate the fold-change of mRNA in testis and *β*-*actin* was taken for internal control. Every sample was measured in three times.

Sf-1-F (5′-TTCTGAGAGCCCGCTAGCCACT-3′),

Sf-1-R (5′-CGTCCGCTGAACGGAAGGAGAA-3′),

*StAR*-F (5′-AAAGCCAGCAGGAGAACGGGGA-3′),

*StAR*-R (5′-GCCTCCATGCGGTCCACAAGTT-3′),

*P450scc*-F: (5′-CTGCCTGGGATGTGATTTTCA-3′)

*P450scc*-R: (5′-GTAATGTTGGCCTGGATGTTCT-3′)

*3β*-*HSD*-F (5′-GCGGCTGCTGCACAGGAATA-3′),

*3β*-*HSD*-R (5′-GACGCATGCCTGCTTCGTGA-3′),

*P450c17*-F (5′-GATCGGTTTATGCCTGAGCG-3′),

*P450c17*-R (5′-TCCGAAGGGCAAATAACTGG-3′),

*17β*-*HSD*-F (5′-GATGTGGCTGTCAACTGTGC-3′),

*17β*-*HSD*-R (5′-TTGATAACCCGCTGGAAGTC-3′),

*β*-*actin*-F (5′-TGGAATCCTGTGGCATCCATGAAAC-3′),

*β*-*actin*-R (5′-TAAAACGCAGCTCAGTAACAGTCCG-3′).

### Western blot

For total protein extraction from the testis, Protein Extraction Kit was used (Merck Millipore, Massachusetts, USA). Protein quantification was carried by the BCA Protein Assay Kit (Merck KGaA, Darmstadt, Germany). The image of SF-1 western blots was got (Additional file 1: Fig. S1) and the operating steps were performed as described earlier [28]. The following primary and secondary antibodies were used: primary rabbit monoclonal anti-actin antibody (1:1000) (Merck Millipore, Darmstadt, Germany, Catalog# 1501), rabbit polyclonal SF-1 antibody (1:200) (Santa Cruz Biotech, Santa Cruz, USA, Catalog# sc-28740), secondary antibody goat anti-rabbit IgG 1:10000 (Thermo Scientific, Rockford, USA).

### Statistical analysis

The data are presented as mean ± standard error in Figures. All data were analyzed by One-way analysis of variance (ANOVA) and the F-test from SPSS 22.0. Difference between groups was considered statistically significant at *p* < 0.05.

## Results

### Content of element Ce in testis after the exposure of CeO_2_ NPs

**Fig. 2:**
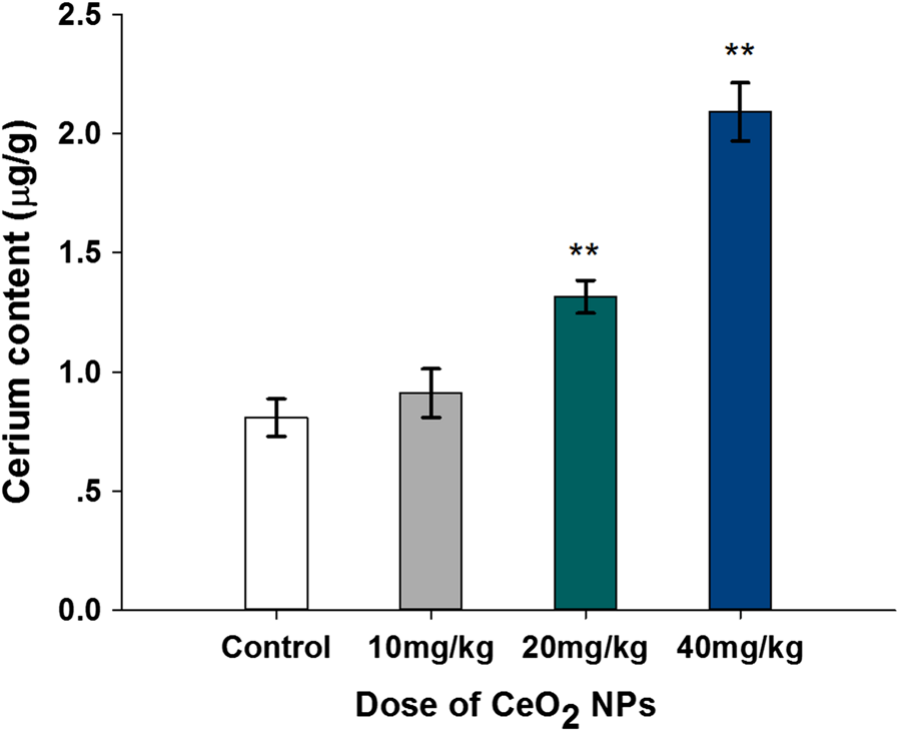
Increased levels of testis element Ce content in mice following the addition of various doses of CeO_**2**_ NPs for 32 days. Values are mean ± SD, *n* = 6. Significance of difference: CeO_**2**_ NPs group compared with control, **p < 0.01

Figure 2 shows an increase of element Ce content in testis after administration of CeO_2_ NPs. There were the most significant differences between 20 or 40 mg/kg doses groups (1.316 or 2.092 μg/g) and in the control group (0.807 μg/g) (*p* < 0.01). The increase of element Ce content in 10 mg/kg dose animal testis was no statistically significant differences (*p* > 0.05).

### Testis weight and Testis-somatic index

**Fig. 3:**
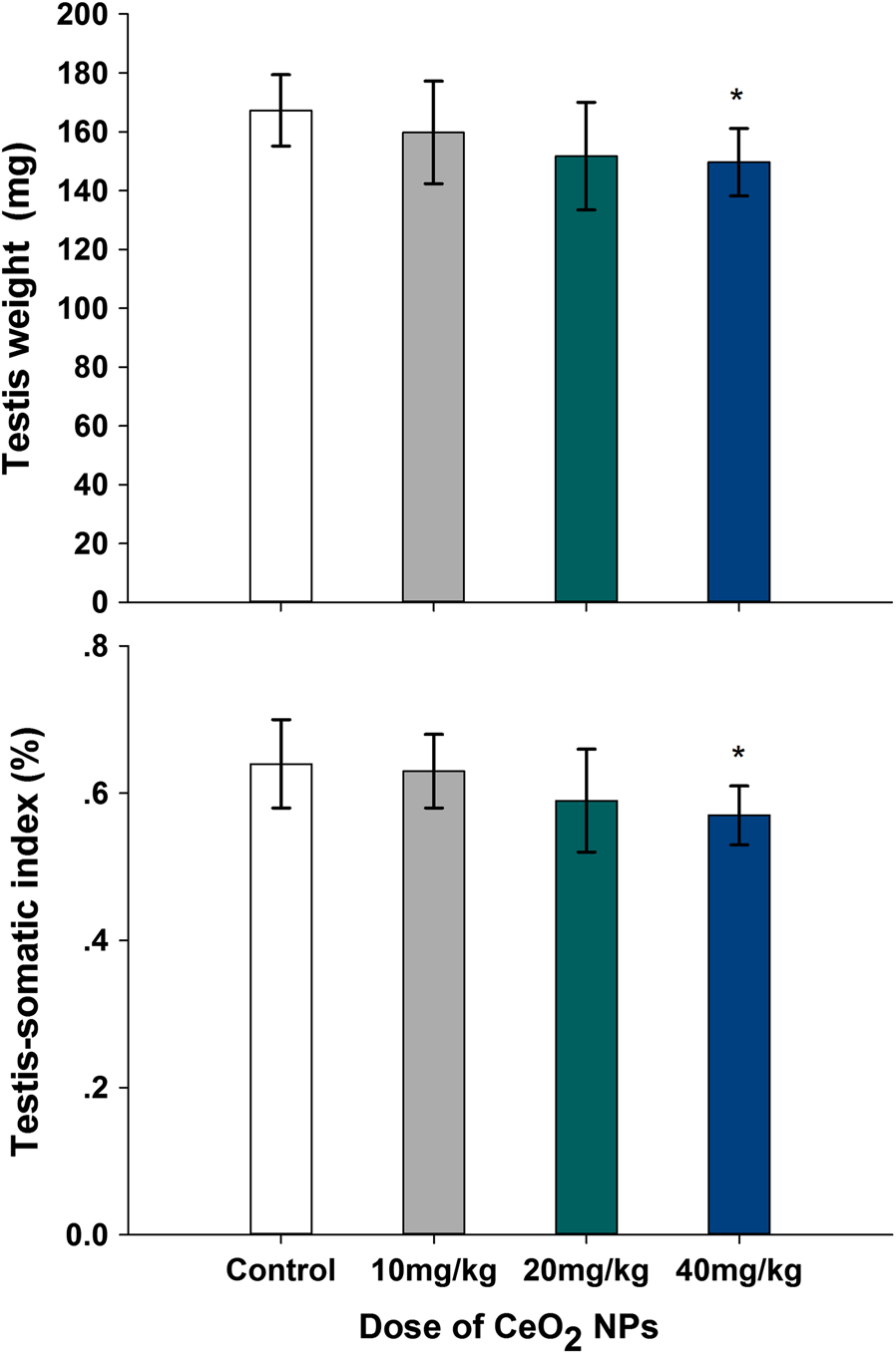
Effects of CeO_2_ NPs on testicular weight in mice following the addition of various doses of CeO_**2**_ NPs for 32 days. Values are mean ± SD, *n* = 12. Significance of difference: CeO_**2**_ NPs group compared with control, *p < 0.05

As shown in Fig. 3a, b, all of testis weight and testis-somatic index had a decrease following CeO_2_ NPs administration. Compared to the control group, the significant difference of testis weight appeared in 40 mg/kg doses groups by 10.54% (*p* < 0.05), Testis-somatic index reduced in 40 mg/kg doses groups from 0.64% to 0.57% (*p* < 0.05).

### DSP and sperm motility

**Fig. 4:**
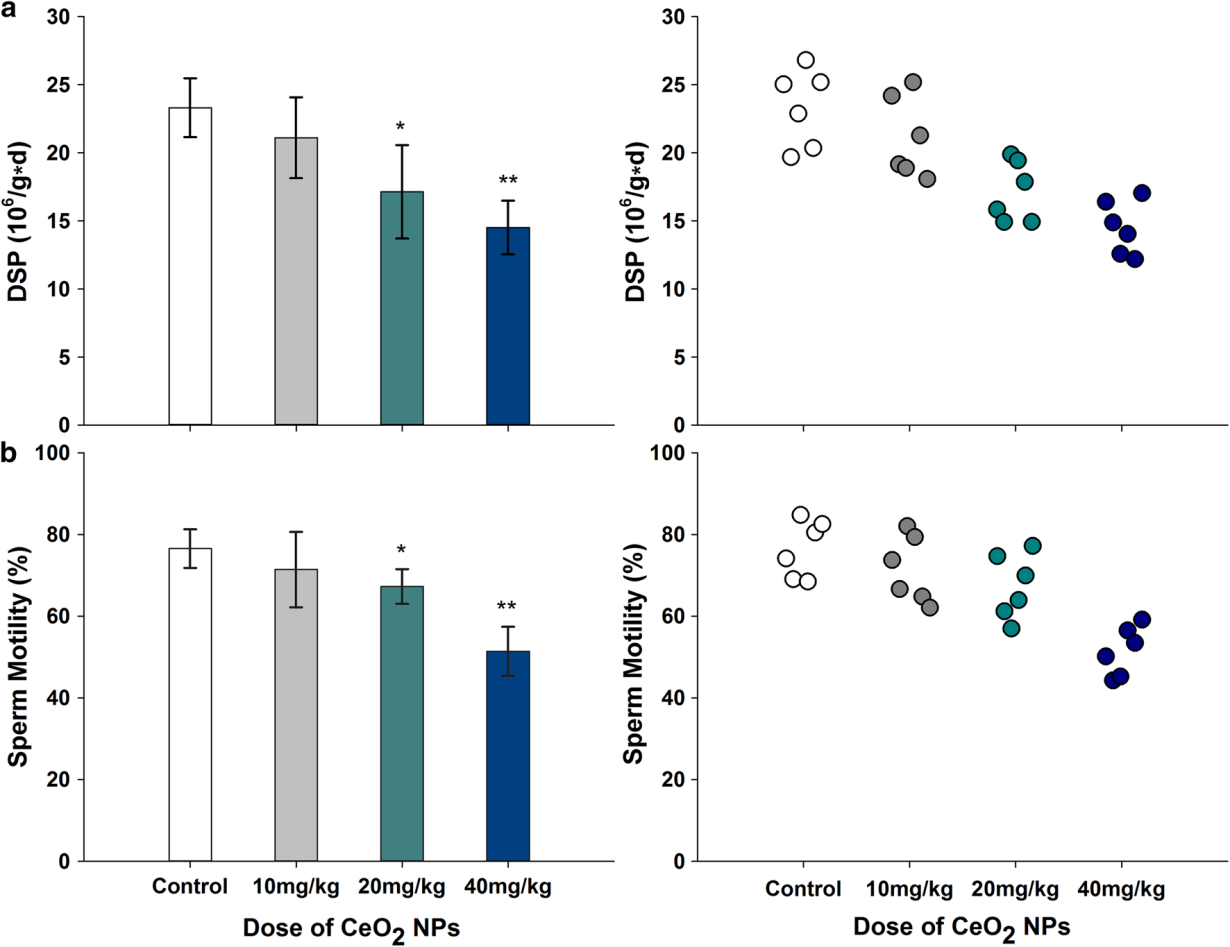
Reduced levels of DSP (**a**) and sperm motility (**b**) following the addition of various doses of CeO_2_ NPs in mice for 32 days. *n* = 6. Significance of difference: CeO_2_ NPs group compared with control, *p < 0.05, **p < 0.01

Oral exposure of mice to CeO_2_ NPs induced significant effect on the testicular DSP and the epididymis sperm motility compared with that of the control group (Fig. 4 a and b). The reductions in DSPs were 26.47% and 37.71% at doses 20 mg/kg and 40 mg/kg, respectively (*p* < 0.05 or *p* < 0.01). A downward trend in sperm motility, 12.10% and 32.84% was also observed in mice given 20 and 40 mg/kg doses, respectively (*p* < 0.05 or *p* < 0.01). However, these alterations in sperm motility and DSP were not significant in mice given 10 mg/kg CeO_2_ (*p* > 0.05).

### Sperm DNA integrity

**Fig. 5:**
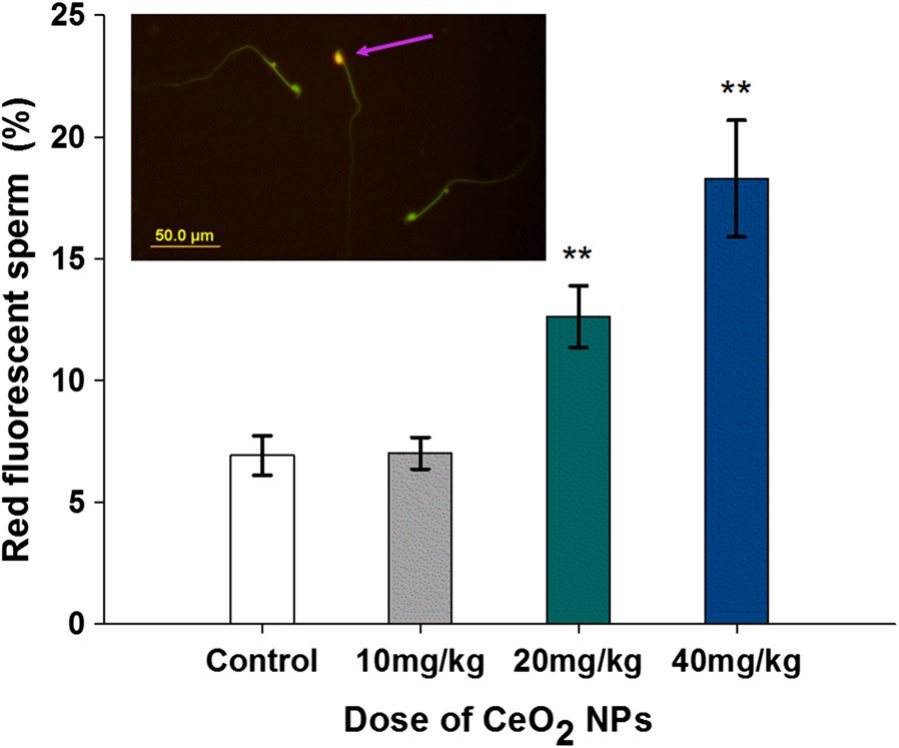
AO staining result for DNA integrity. The green and red fluorescence indicate the sperm with normal double-stranded DNA and the abnormally denatured DNA, respectively. Arrow shows the sperm head appeared with red fluorescent. Column diagram indicates percentage of red fluorescent labeled sperm in mice exposed to various doses CeO_2_ NPs. Values are mean ± SD, *n* = 12. Significance of difference: CeO_2_ NPs group compared with control, *p < 0.05, **p < 0.01

The AO staining analysis revealed oral exposure CeO_2_ NPs in mice resulted in a concentration-dependent decrease in the epididymal sperm DNA integrity. The native DNA (green fluorescence) and denatured DNA (red fluorescence) in sperm head are showed in Fig. 5. Red fluorescence sperm ratio which represents denatured sperm DNA in 20 and 40 mg/kg CeO_2_ NPs groups were increased significantly compared to the control from 6.93% to 12.62% or 18.29% (*p* < 0.01). Hence, it was considered that the sperm DNA was highly susceptible to CeO_2_ NPs exposure.

### Testicular histology

**Fig. 6:**
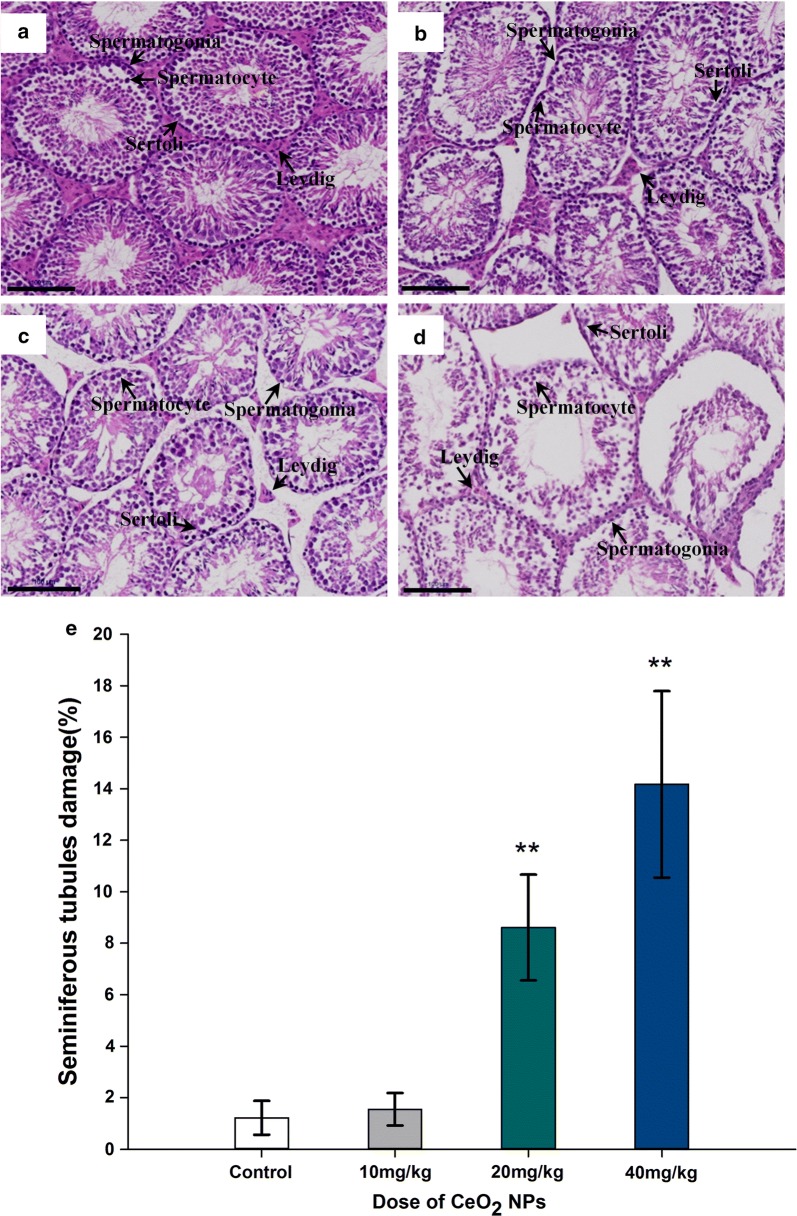
Histopathological changes in the testis tissues. **a**–**d** Photomicrograph of transverse section of H&E staining of mice in control, CeO_2_ NPs groups (10, 20 and 40 mg/kg), Bar length = 100 microns. **e** The percentage of degenerative seminiferous tubules in testicular cross sections. Values are mean ± SD, *n* = 6. Significance of difference: CeO_2_ NPs group compared with control, *p < 0.05, **p < 0.01

At the end of the CeO_2_ NPs exposure, there were degenerative changes in testis tissue (atrophy of seminiferous tubules or necrosis, the seminiferous epithelium cellular adhesion loosening or desquamation, spermatozoa loss and interstitial tissue apoptosis) in mice given 20 and 40 mg/kg CeO_2_ NPs compared to the control group, (Fig. 6). From the histological studies, a distinct decline was presented in the several dominant cell types such as the Leydig cells, Sertoli cells, spermatogonia, primary spermatocytes and spermatids. From the tubular cross-sections, the ratio of seminiferous tubule damage in the CeO_2_ NPs 20 and 40 mg/kg groups were significantly increased compared to control group (*p* < 0.01).

### Testicular enzyme assay

**Fig. 7:**
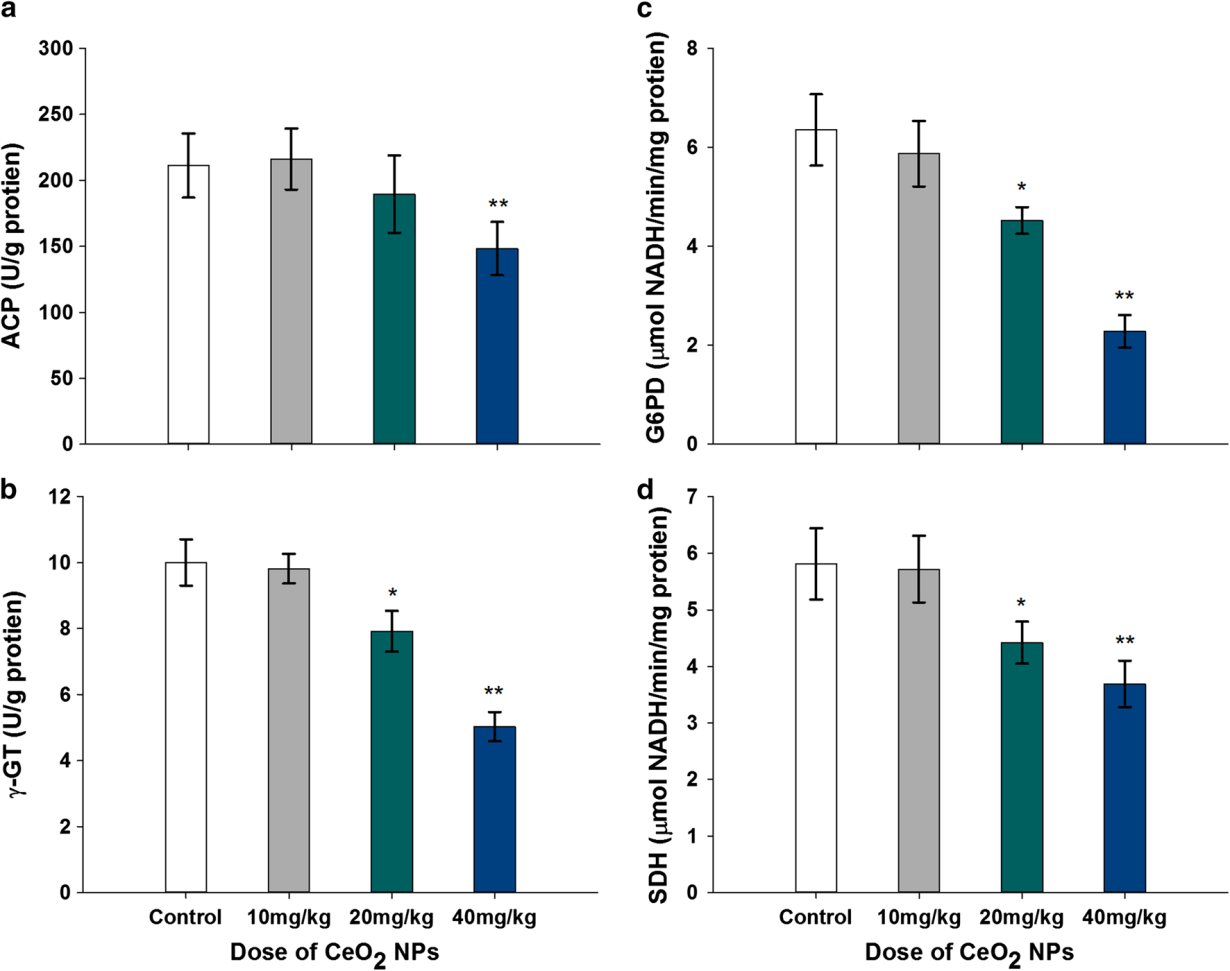
Changed activities’ levels of testicular marker enzymes, ACP (**a**), γ -GT (**b**), G6PD (**c**) and SDH (**d**) in mice following the addition of various doses of CeO_**2**_ NPs for 32 days. Values are mean ± SD, *n* = 6. Significance of difference: CeO_**2**_ NPs group compared with control, *p < 0.05, **p < 0.01

The activities of testicular marker enzymes which are known to be associated with testicular function were changed after CeO_2_ NPs exposure. The activities of G6PD, γ-GT, and SDH in mice given 20 mg/kg CeO_2_ NPs were decreased significantly, 28.82%, 20.88%, and 23.92%, respectively, compared to the control group (*p* < 0.05). In the 40 mg/kg CeO_2_ NPs treated animals, the results indicated significant decrease in four testicular marker enzymes activities (ACP, G6PD, γ -GT, and SDH) by 29.81%, 64.09%, 49.75%, and 36.49% (*p* < 0.01), respectively. The activity of G6PD was significantly reduced in the four testicular marker enzymes (Fig. 7)

### Plasma testosterone level (nmol/L)

**Fig. 8:**
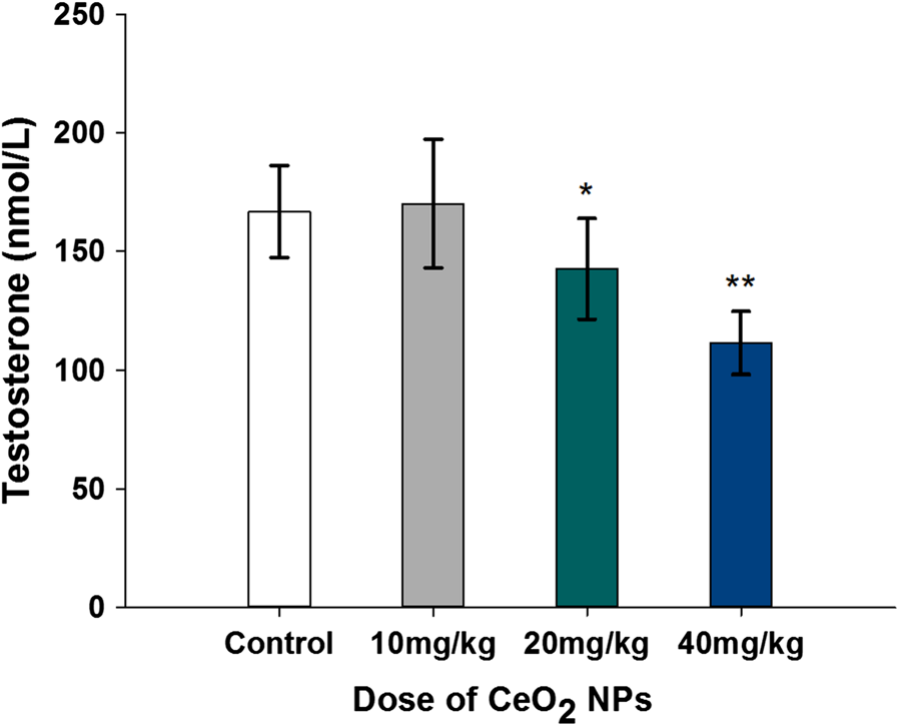
Reduced levels of plasma testosterone in mice following the addition of various doses of CeO_**2**_ NPs for 32 days. Values are mean ± SD, *n* = 12. Significance of difference: CeO_**2**_ NPs group compared with control, *p < 0.05, **p < 0.01

The administration of CeO_2_ NPs significantly decreased plasma testosterone concentration (Fig. 8). CeO_2_ NPs exposure in 20 mg/kg and 40 mg/kg groups decreased the testosterone levels by 14.46% and 33.17%, respectively, relative to the control mice (*p* < 0.05 or *p* < 0.01).

### Testosterone genes expressions

**Fig. 9:**
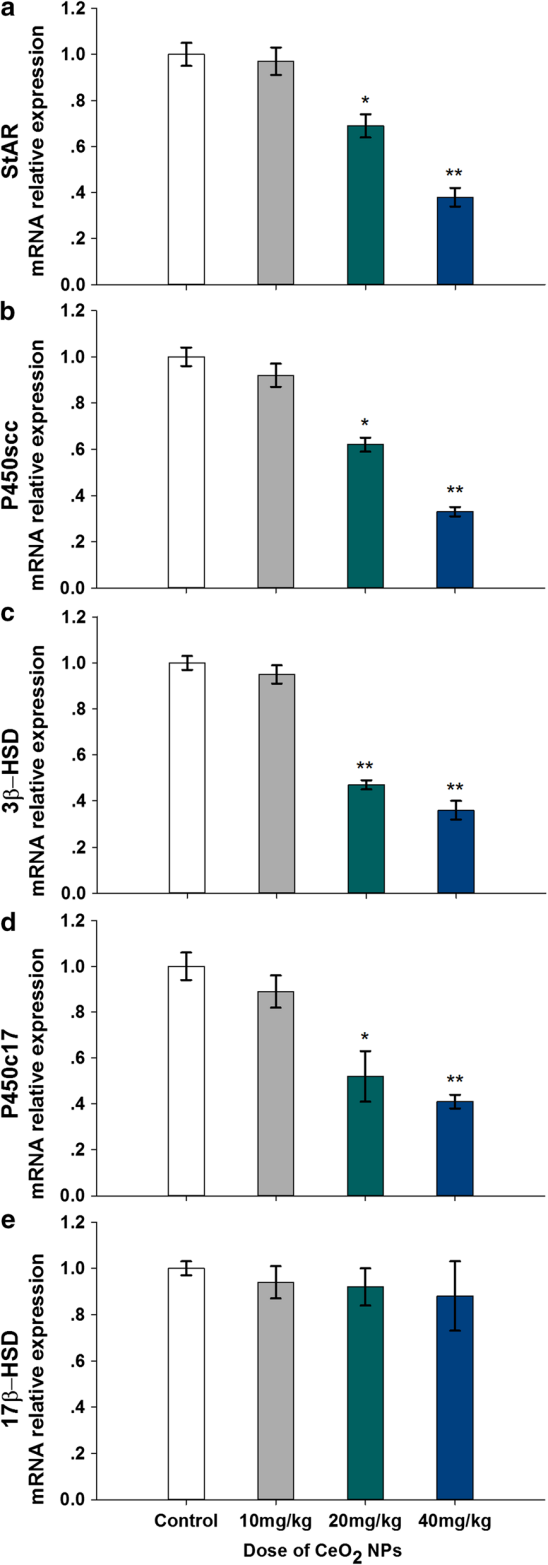
Changed expression levels of testosterone synthetic genes, *StAR* (**a**), *P450scc* (**b**), *3β*-*HSD* (**c**), *P450c17* (**d**) and *17β*-*HSD* (**e**) in testes of mice following the addition of various doses of CeO_**2**_ NPs for 32 days. Values are mean ± SD, *n* = 6. Significance of difference: CeO_**2**_ NPs group compared with control, * *p* < 0.05, ** *p* < 0.01

The real-time PCR results of five testosterone gene expressions (*StAR, P450scc, 3β*-*HSD, P450c17, 17β*-*HSD*) are presented in Fig. 9a–e, respectively. Compared with expression levels of the five genes in control animals’ testis, these data indicated that the CeO_2_ NPs exposure induced a significant and dose-dependent decrease in 20 and 40 mg/kg dose animals (*p* < 0.05 or *p* < 0.01) except 17β-HSD (*p* > 0.05).

### SF-1 expressions

**Fig. 10:**
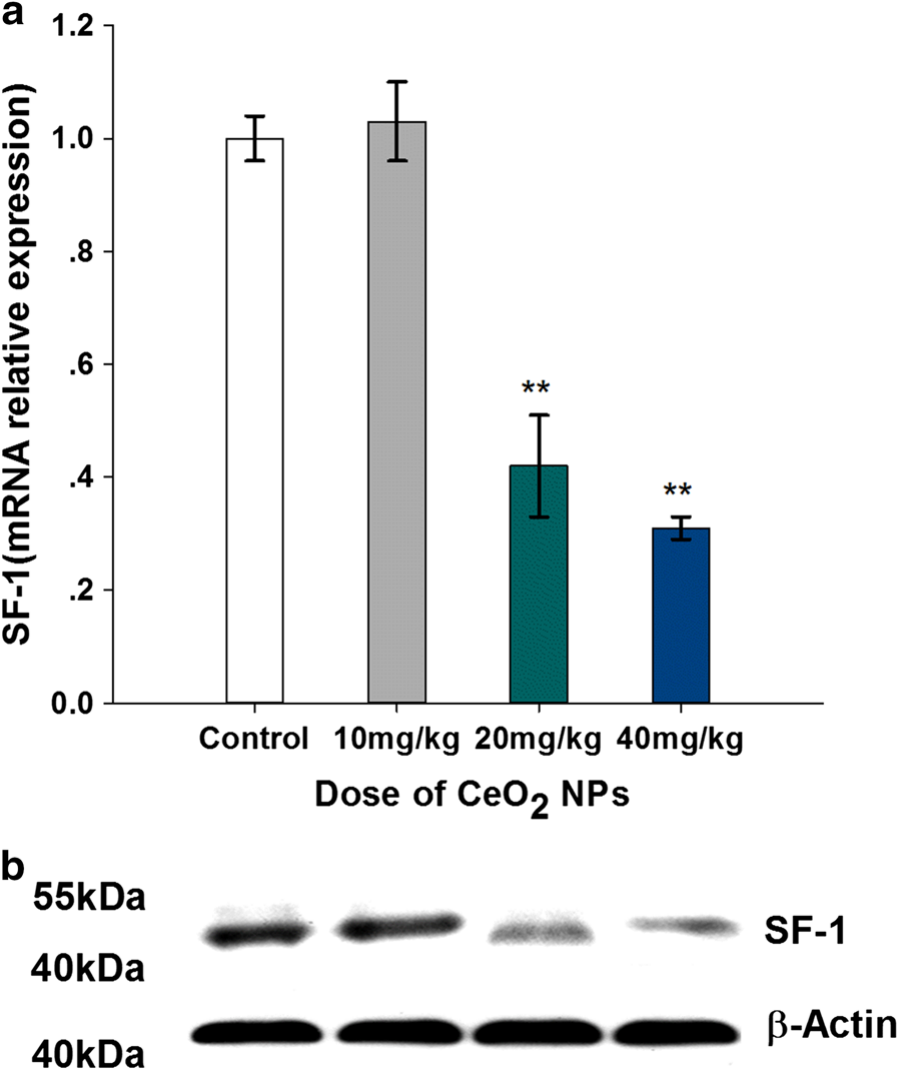
Changed mRNA (**a**) and protein (**b**) expression levels of steroidogenic factor-1 in testes of mice following the addition of various doses of CeO_2_ NPs for 32 days. Values are mean ± SD, *n* = 6. Significance of difference: CeO_2_ NPs group compared with control, **p < 0.01

The real-time PCR and Western Blot results of *Sf*-*1* are presented in Fig. 10a, b, respectively. Compared with control group, mRNA expression levels of *Sf*-*1* gene were significantly down-regulated by 0.58 and 0.69-fold in CeO_2_ NPs exposed mice at 20 and 40 mg/kg, respectively. Similar effects of CeO_2_ NPs on SF-1 protein expression were also observed. Therefore, CeO_2_ NPs exposure down-regulated the expression levels of transcription factor SF-1 in mice testis.

## Discussion

Successful reproduction may not be possible in the absence of integrity in male gamete, and impairment of sperm functions can lead to abnormal offsprings [29]. Among the methods in assessment of toxic effects on spermatogenesis, enumeration of testicular sperm head has been widely used as a simple, reproducible, and quantifiable one [30]. Daily sperm production (DSP) is also a common method in detection of testicular toxicity [31]. In the present study, both the methods were used and the results showed that DSP decreased significantly in the 20 and 40 mg/kg CeO_2_ NPs group, but not in the 10 mg/kg group, indicating a high dose effect of CeO_2_ NPs on the spermatogenesis process. While the increased element Ce content of testis in nano cerium oxide treatment animals reveal the cerium oxide nanoparticles enter into the testicle and accumulated. The experiment results indicated the toxicity of spermatogenesis induced by high dose CeO_2_ NPs be correspondence to the increased element Ce in testicle.

As one of the major determinants of male fertility, the sperm motility reflects its ability of penetration and thus of male reproduction [32]. El-Sabeawy et al. reported that exposure to some toxicants may decrease testicular sperm numbers and sperm motility, and depress acrosome reactions [33]. In the present study, sperm motility was significantly decreased with increasing CeO_2_ NPs dose. In addition to parameters such as sperm number, concentration, motility and morphology, another important key marker for sperm quality is chromatin integrity which directly affects reproduction procedure such as fertilization, embryo development, and pregnancy outcome [34]. In this study, AO staining test revealed that the percentage of sperm with single stranded DNA increased significantly in 20 and 40 mg/kg CeO_2_ NPs groups, indicating an impairment of the DNA integrity. The reduction in sperm motility induced by CeO_2_ NPs may be due to its damage to sperm cells in epididymis and/or in testicular, since histopathological examination revealed that CeO_2_ NPs induced a degeneration of spermatids in testicular tissue.

The microscopic examination of testis tissue sections confirmed that CeO_2_ NPs exposure induced degenerative changes in 20 and 40 mg/kg groups, such as atrophy/necrosis of seminiferous tubules and apoptosis in interstitial tissue. Earlier studies reported that some enzymes played important roles in the functional status of specific cell type in testis during germ cell maturation [35, 36]. Among the testicular enzymes, SDH is relevant to germ cell maturation [37], γ-GT parallels the maturation and replication of Sertoli cells and, ACP’s activity reveals the Sertoli cell function [38]. In spermatogonial cells such as primary/secondary spermatocytes, spermatids and spermatozoon bundles, the advent of ACP may provide phosphate to meet high energy requirements [39]. Activities of γ-GT, ACP and SDH were decreased upon CeO_2_ NPs exposure. Besides, G6PDH in Leydig cells were also changed in mice given oral 20 and 40 mg/kg, which would impair testosterone production.

Previous investigations suggested that pathological changes in testes induced by toxic chemicals could alter imbalance in production of sex hormones [40]. Testosterone is known to be indispensable for spermatogenesis and its concentration in the seminiferous tubules is generally 10–100 times higher than that in blood [41, 42]. Through androgen receptor, testosterone functions on spermatogenesis by acting on Sertoli cells to provide nutritional and morphogenetic support for germ cells [43, 44]. When entering into the epididymis through efferent ducts, testosterone helps sperm in obtaining progressive motility and fertilization capabilities during maturation [45]. In this study, 20 and 40 mg/kg CeO_2_ NPs significantly decreased testosterone concentrations in plasma, indicating the potential that lack of testosterone may lead to disorders in spermatogenesis and maturation and therefore infertility [46].

Testosterone is mainly produced in the Leydig cells in testis by a series of enzymatic reactions. As the first step, the mitochondrial cytochrome P450scc enzyme system transforms cholesterol to pregnenolone [47, 48]. Then, other testosterone synthesis enzymes (3β-HSD, P450c17, 17β-HSD) convert the pregnenolone to testosterone [49]. For testosterone synthesis, transferring of cholesterol to mitochondria requires steroidogenic acute regulatory protein (StAR) which is considered to be the rate-limiting step in steroid biosynthesis [50]. At the gene expression level, the function of Leydig cells is largely dependent upon the genes coding StAR protein, cytochrome P450cc and P450c17, HSD3β and HSD17β. In the present study, the genes coding these enzymes were significantly down-regulated, which may result in reduced synthesis of male hormone and impaired maturation of sperm.

The genes expression encoding these enzymes is tightly under the control of a battery of transcription factors in the testes [51]. The expression of key protein StAR that plays an essential role in testosterone synthesis was reported to be regulated by steroidogenic factor-1 (SF-1, NR5A1, Ad4BP) [52], which is a nuclear receptor regulatory gene involved in steroidogenesis, adrenal/gonadal development and the reproductive axis [53]. The investigation into human infertility cases from Bashamboo and co-workers found that the men who harbored *NR5A1* changes had lower levels of testosterone and more fateful infertility such as azoospermia and oligozoospermia [54]. SF-1 has been reported to increase expression of the machinery of steroid biosynthesis by binding to its response element site in the promoter regions of the genes encoding for *StAR*, Hydroxysteroid dehydrogenases (*3β*-*HSD*, *17β*-*HSD*) and cytochrome P450 steroid hydroxylase (CYP) enzymes including *P450scc* and *P450c17* [55–57]. In testosterone biosynthesis, P450c17 converts pregnenolone to dehydroepiandrostenedione (DHEA) in the sex steroid pathways. SF-1 activates transcription of the P450c17 gene whose promoter containing three functional SF-1 sites through multiple cis-elements [58, 59]. In SF-1 knockout mice, hypoplastic testes were observed with the obstacle of spermatogonia development into mature sperm, together with markedly decreased expression of P450scc and StAR, two essential components of testosterone biosynthesis in leydig cell [60–62]. In this study, the reduced gene expression levels of testosterone synthesis gene (*StAR, P450scc, P450c17, 3β*-*HSD* and *17β*-*HSD*) induced by CeO_2_ NPs exposure was correlated with down-regulated mRNA and protein levels of SF-1 gene expression. This correlation prompts a possibility of SF-1 as a mediator of reduced testosterone synthesis by CeO_2_ NPs treatment, but further tests are needed.

It is worth noting that the results of CeO_2_ NPs treatment will exhibit different effects to the sperm quality in male animals due to dose, nanoparticle size, animals’ kinds, administration methods such as peroral (PO), intravenous (IV), and intraperitoneal (IP). The data changes in the present study agree with a report in mice administrated with CeO_2_NPs via intraperitoneal (IP) route by 100–300 μg/kg BW thrice/week for 35 days [63]. Although another literature reported that the CeO_2_ NPs administration (30 mg/kg, IP) for fourteen days can ameliorate sperm and testicular damage induced by diabetes in rats, it alone still distinctly reduced the leydig cell density, sertoli cell density, inner and outer diameter of seminiferous tubule in testis [64]. Therefore, the results obtained in the present study exhibited the peroral (PO) effect of CeO_2_ NPs exposure on male mice reproductive toxicity for consecutive 32 days. Meanwhile, the results from this study present the public with precaution for reasonable application of cerium oxide nanomaterial.

## Conclusion

In this study, long-term, oral administration of CeO_2_ NPs at doses over 20 mg/kg could impair male reproductive functions by decreasing sperm count and motility, destructing DNA integrity and disturbing testosterone synthesis with down-regulation of transcription regulation factor SF-1. This work hints that the utilization of CeO_2_ NPs needs to be carefully evaluated about their potential reproductive toxicity on the human health.

## Additional file

Additional file 1: Fig. S1. Original image of SF-1 Western blots.
