# Supplementary figures and images for: SF-1 mediates reproductive toxicity induced by Cerium oxide nanoparticles in male mice
Source: J Nanobiotechnology. 2019 Mar 21;17:41. doi: 10.1186/s12951-019-0474-2 (PMC6427857; doi:10.1186/s12951-019-0474-2)

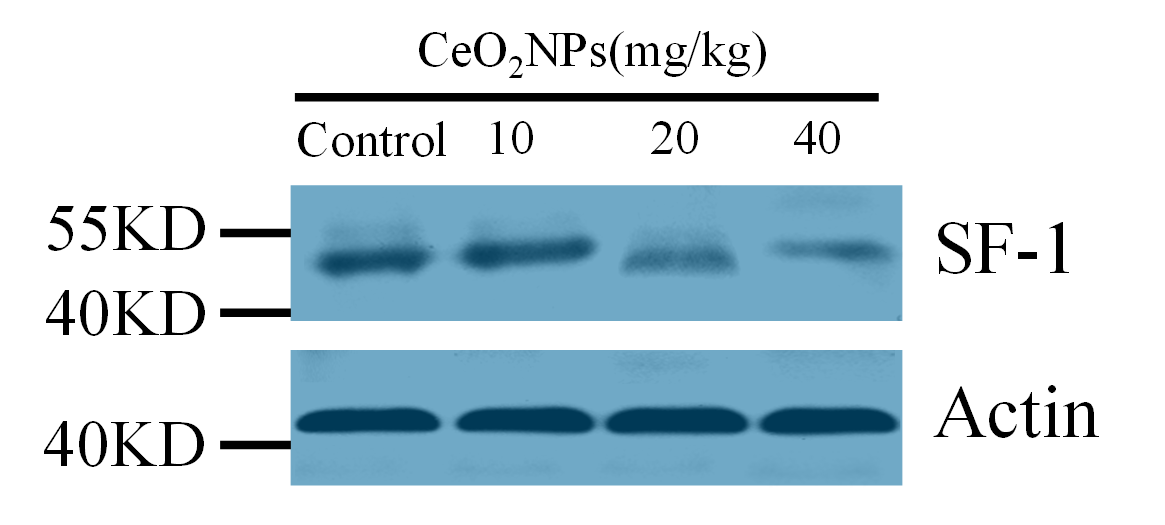

Supplement: Supplementary file 1 — Additional file 1: Fig. S1. Original image of SF-1 Western blots. [file 12951_2019_474_MOESM1_ESM.tif]
